# Duration of treatment in oncology clinical trials: does the duration change when the same drug moves from the experimental arm to the control arm?

**DOI:** 10.1016/j.esmoop.2022.100480

**Published:** 2022-04-22

**Authors:** A. Haslam, T. Olivier, R. Thawani, V. Prasad

**Affiliations:** 1 Department of Epidemiology and Biostatistics, University of California San Francisco, San Francisco, USA; 2 Department of Oncology, Geneva University Hospital, Geneva, Switzerland; 3 Division of Hematology and Oncology, Oregon Health and Science University, Portland, USA

**Keywords:** duration of treatment, progression-free survival, clinical trial, drug approval, comparator, intervention

## Abstract

**Background:**

When a new drug comes to the market, the incentive for the sponsoring company is to maximize the treatment duration in order for the patient to reap the full therapeutic benefit of the product and achieve a positive trial result. We sought to enumerate instances when an already-approved oncology drug was used as a comparator for a newer drug seeking approval and compare the duration of treatment when it is used in the intervention arm to when it is used as a comparator.

**Patients and methods:**

In a cross-sectional analysis, we searched drug approval announcements for advanced, metastatic, or unresectable cancers between 2009 and 2020. We included studies reporting on an approved drug and studies reporting on when the same drug was used as a comparator for other drugs seeking Food and Drug Administration (FDA) approval. We examined median progression-free survival and duration of treatment for when the drug was initially approved and for when the drug was used as a comparator for other drugs that were seeking approval.

**Results:**

Of the 23 instances when an approved drug was later used as a comparator against a newer drug seeking FDA approval, we found 11 instances (47.8%) where the drug, when used as a comparator arm, had a shorter duration of treatment than when it was used in the intervention arm. The median duration of treatment in the study initially testing the drug was 6.0 months (range: 2.2-12.7 months), whereas the median duration of treatment when the same drug was used as a comparator was 4.9 months (range: 1.7-12.0 months).

**Conclusions:**

These results suggest that there is bias in how long a patient receives a given therapy, and this bias favors the newer therapy. Clinical trialists should seek to utilize methodology that reduces bias so that the relative efficacy of newer drugs can be objectively assessed.

## Highlights

• We found 23 instances when an approved drug was later used as a comparator against a newer drug seeking FDA approval.
• Drugs, as a comparator, had a shorter duration of treatment than when used in the intervention arm in 48% of instances.
• The median durations of treatment for drugs seeking approval and then as comparators were 6 and 5 months, respectively.

## Introduction

Over the past few decades, treatment options for patients with cancer have expanded, which has benefited patients in the form of longer time to tumor progression and overall survival. The shift away from cytotoxic therapies to targeted therapies, including monoclonal antibodies, has also led to an ∼75% increase in the duration of treatment for the patient, in part because of better tolerability of the drug.^1^ With the better tolerability of newer drugs, the continuation of treatment beyond progression is being increasingly used in an effort to slow further progression.^2^^,^^3^ However, regarding some classes of chronic treatment strategies, such as immune checkpoint inhibitors, long-term effects on the immune system are still unknown prompting others to caution against unnecessary lengthy durations of treatment.

When a new drug comes to the market, the incentive for the sponsoring company is to maximize the treatment duration in order for the patient to reap the full therapeutic benefit of the product and achieve a positive trial result.^4^ As next-in-class drugs or alternative therapies enter the marketplace, they may be tested against the older, established product. Ideally, the established therapy in these trials will be given at least as long as in the initial pivotal study, or arguably even longer, as physicians become more comfortable managing the unique toxicity profile of the agent and encourage responding patients to continue the drug. Unfortunately, however, the new sponsoring company may have a perverse incentive: the less time patients take the established drug in the control arm, the greater the likelihood of demonstrating therapeutic superiority of their new product. We sought to investigate if this bias was present in cancer randomized trials, and if so, whether it occurred preferentially for some disease indications or drug classes.

We sought to identify and characterize situations where an approved drug was used as a comparator drug against a newer drug seeking Food and Drug Administration (FDA) approval at a later date. Specifically, we calculated the duration of treatment and quantified the frequency when an already-approved drug had a shorter duration of treatment when used as a comparator, compared to the duration of treatment when the same drug was previously tested as the ‘new’ drug.

## Methods

We searched all FDA announcements for anticancer drug approvals for advanced, metastatic, or unresectable cancers between 2009 and 2020. We used FDA announcements because this provided us with a systematic list of drugs to compare, and we chose those dates because we were able to assemble a comprehensive list of FDA drug approvals going back to 2009 with the help of a previous analysis.^5^ For all drug approvals that met our criteria, we searched for the pivotal trials that reported data used for the FDA approval. From the published trial data, we abstracted information about the median progression-free survival (PFS) for the control and intervention arms, the median duration of treatment for each arm, the indication, the drug used in the comparator arm if not placebo, and the date of approval. If the median number of cycles was reported instead of the duration of treatment, we calculated the median duration of treatment by using the duration of each cycle. For drugs that were approved in 2009 or later but used a comparator drug that was approved before 2009, we searched for the published study that reported on the trial results used for its approval and abstracted trial data.

For studies that reported both the median duration of treatment and median PFS, we plotted these values and calculated a regression line for both intervention and control arms. Using these regression slopes, we then estimated the duration of treatment for studies that reported median PFS but not median duration of treatment.

We then looked for all indications where a drug was approved and then was later used as a comparator in other FDA approvals. We calculated the median PFS and duration of treatment for trials testing drugs for initial approval (original study) and for trials that used the same drug as a comparator for other drugs seeking approval. We calculated the number of times the duration of treatment was longer and shorter for the initial approval than when the same drug was used as a comparator.

We used Microsoft Excel^6^ for these analyses, and calculated frequencies (percentages) and medians (ranges). In accordance with United States Health and Human Services regulations 45 CFR §46.102(f), this study was not submitted for institutional review board approval because it involved publicly available data and did not involve individual patient data.

## Results

We found 171 unique approvals between 2009 and 2020, and we found eight drugs approved before 2009 that were used as a comparator for drugs approved between 2009 and 2020, for a total of 179 approvals included in our analysis. Most drugs were tested against a placebo or was a drug tested in combination with an existing drug or drug combination against the same drug or drug combination without the novel drug (55%; *n* = 99/179).

**Figure 1:**
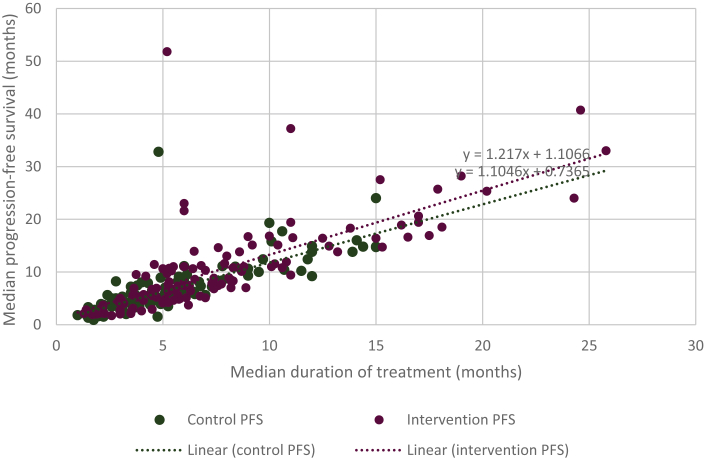
**Correlation between median progression-free survival and duration of treatment for** US **Food and Drug Administration approv**ed drugs in the advanced or metastatic setting (2009-2020)**.** PFS, progression-free survival.

For the intervention group, we calculated a regression line of 1.217x + 1.11066, and for the control group, we calculated a regression line of 1.1046x + 0.7365 (Figure 1). We noted a linear relationship between the median PFS and median duration of treatment for when drugs were used as interventions and controls, and we observed that the slope was similar in both instances (1.2 versus 1.1). Using these regression lines, we imputed the duration of treatment for 36 (20%) control values and 29 (16%) intervention values.

For two drugs that were later used as comparators, we were not able to determine duration of treatment when the drug was initially tested, either because of very high overall survival rate (e.g. imatinib for chronic myeloid leukemia) or because neither the duration of treatment nor PFS data were reported (e.g. dacarbazine for melanoma); thus we were not able to use these drugs in our analysis.

**Figure 2:**
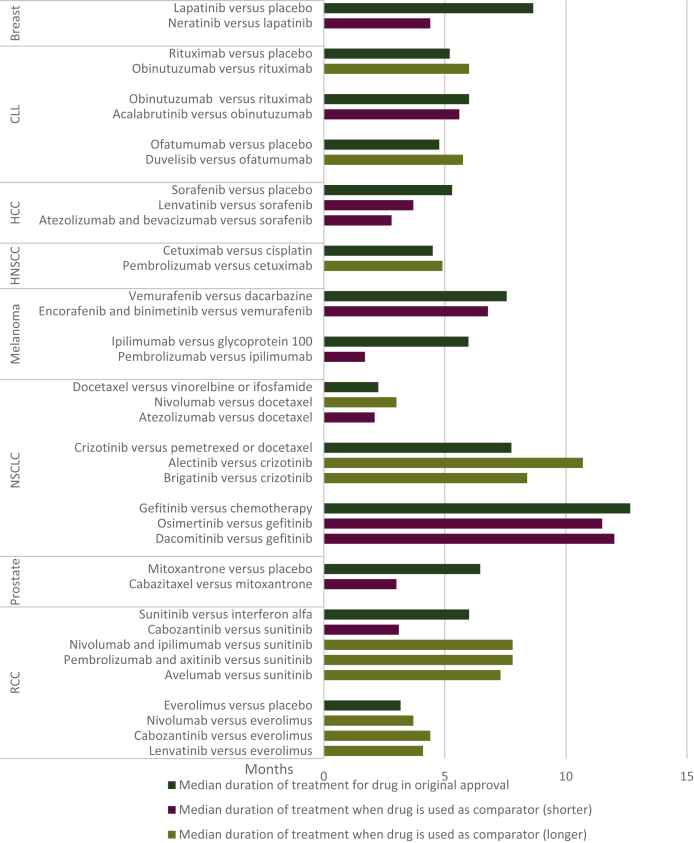
**Median duration of treatment for approved drugs in trials testing them and when they are used as a comparator drug against a newer drug seeking Food and Drug Administration approval at a later date.** CLL, chronic lymphocytic leukemia; HCC, hepatocellular carcinoma; HNSCC, head and neck squamous cell carcinoma; NSCLC, non-small-cell carcinoma; RCC, renal cell carcinoma.

We found eight tumor types and 13 different indications, for a total of 23 instances (out of 179; 10 instances being for duplicate indication), where a previously FDA-approved drug was later used as a comparator for the same indication (Table 1; Figure 2). For each drug/indication combination, between one and four drug approvals were given using the same drug as a comparator (four indications where two drugs were given approval, two indications where three drugs were given approval, and one indication where four drugs were given approval). One drug was used as both an novel drug and a comparator drug. When the drug was originally seeking approval, there were 4 (of 14; 28%) times when the trial for approval was blinded. When the drug was used as a comparator, there was 1 (of 23; 4%) time when the trial was blinded.

**Table 1:** Duration of treatment times for drugs that were FDA approved (through 2020) and later used as a comparator for another FDA-approved drug, using the same indication

Tumor	Specific indication	Drug comparison	Median progression-free survival, months	Median duration of treatment for the intervention drug, months	Median duration of treatment when used as a comparator, months	Length of duration of treatment compared to when used as the intervention drug
**Breast**	Previously treated HER2 breast cancer	Lapatinib versus placebo	6.2	8.6		
		Neratinib versus lapatinib	5.5		4.4	Shorter
**CLL**	Untreated	Rituximab versus placebo	32.8	5.2		
		Obinutuzumab versus rituximab	11.1		6.0	Longer
	Untreated	Obinutuzumab versus rituximab	23.0	6.0		
		Acalabrutinib versus obinutuzumab	22.6		5.6	Shorter
**CLL**	Recurrent/relapsed	Ofatumumab versus placebo	18.8	4.8		
		Duvelisib versus ofatumumab	9.1		5.8	Longer
**HCC**	First line	Sorafenib versus placebo	10.7	5.3		
		Lenvatinib capsules versus sorafenib	3.6		3.7	Shorter
		Atezolizumab in combination with bevacizumab versus sorafenib	4.3		2.8	Shorter
**HNSCC**	First line	Cetuximab versus cisplatin	5.5	4.5		
		Pembrolizumab versus cetuximab	5.1		4.9	Longer
**Melanoma**	BRAF V600E mutation	Vemurafenib versus dacarbazine	5.3	7.6		
		Encorafenib and binimetinib versus vemurafenib	7.3		6. 8	Shorter
**Melanoma**	First line	Ipilimumab versus glycoprotein 100	4.0	6.0		
		Pembrolizumab versus ipilimumab	2.8		1.7	Shorter
**NSCLC**	Second line	Docetaxel versus vinorelbine or ifosfamide	2.0	2.2		
		Nivolumab versus docetaxel	2.3		3.0	Longer
		Atezolizumab versus docetaxel	3.6		2.1	Shorter
	ALK+	Crizotinib versus pemetrexed or docetaxel	7.7	7.8		
		Alectinib versus crizotinib	10.4		10.7	Longer
		Brigatinib versus crizotinib	11.0		8.4	Longer
	EGFR 19/21	Gefitinib versus chemotherapy	9.5	12.7		
		Osimertinib versus gefitinib	10.2		11.5	Shorter
		Dacomitinib versus gefitinib	9.2		12.0	Shorter
**Prostate**	Advanced hormone-refractory prostate cancer	Mitoxantrone versus placebo	4.4	6. 5		
		Cabazitaxel versus mitoxantrone	1.4		3.0	Shorter
**RCC**	First line	Sunitinib versus interferon alfa	11.0	6.0		
		Cabozantinib versus sunitinib	5.3		3.1	Shorter
		Nivolumab and ipilimumab versus sunitinib	8.4		7.8	Longer
		Pembrolizumab plus axitinib versus sunitinib	11.1		7.8	Longer
		Avelumab versus sunitinib	7.2		7.3	Longer
	Second line	Everolimus versus placebo	4.0	3.2		
		Nivolumab versus everolimus	4.4		3.7	Longer
		Cabozantinib versus everolimus	3.8		4.4	Longer
		Lenvatinib versus everolimus	5.5		4.1	Longer

ALK, anaplastic lymphoma kinase; CLL, chronic lymphocytic leukemia; EGFR, epidermal growth factor receptor; FDA, Food and Drug Administration; HCC, hepatocellular carcinoma; HER2, human epidermal growth factor receptor 2; HNSCC, head and neck squamous cell carcinoma; NSCLC, non-small cell carcinoma; RCC, renal cell carcinoma.

The median duration of treatment in the study initially testing the drug was 6.0 months (range: 2.2-12.7 months), whereas the median duration of treatment when the same drug was used as a comparator was 4.9 months (range: 1.7-12.0 months). The median PFS for the drug in the original study was 7.0 months (range: 2.0-32.8 months), whereas the median PFS when the same drug was used as a comparator was 5.5 months (range: 1.4-22.6 months).

Of the 23 instances when an approved drug was later used as a comparator against a newer drug seeking FDA approval, we found 11 instances (47.8%) where the drug, when used as a comparator arm, had a shorter duration of treatment than when it was used in the intervention arm. We found 12 instances (52.2%) where the drug, when used as the intervention drug, had a longer duration of treatment than when it was used as a comparator.

The percentage of studies that had independently assessed PFS was 67% (*n* = 8/12) when the drug had a longer duration of treatment as a comparator and 73% (*n* = 8/11) when the duration of treatment was shorter. The percentage of studies that were blinded was 0% (*n* = 0/12) when the duration of treatment was longer as a comparator and 9% (*n* = 1/11) when the duration of treatment was shorter.

## Discussion

We found that in about half of approved drugs that were later used as a comparator against a newer drug seeking FDA approval, the duration of treatment of the drug when used as a comparator drug was shorter than its duration of treatment when initially tested. These findings suggest that there is concern for how drugs are being prioritized in clinical studies. When drugs are first approved, there is incentive for trialists to push these drugs, but later, when these same drugs are used as comparators, the incentive then switches to the new drug being investigated. Study trial designs such as open-label, which were more common in studies where the drug of interest was the comparator, have the potential to worsen this bias. Differences in drug-dosing adaptation rules may also play a role in pushing the experimental arm.^4^

We see evidence for this in the median PFS and duration of treatment, where the median PFS for the same drug/indication is shorter when the drug is used as a comparator than when it was originally studied, and the duration of treatment is shorter when the drug is being used as a comparator. These discrepancies suggest that there is bias in how long a patient in a clinical trial is left on a given treatment, favoring the newer drug.

Other researchers have observed a 53% decrease in duration of treatment over time for patients in sorafenib for hepatocellular cancer trials, although they found that PFS among the analyzed trials remained stable and overall survival improved over time.^7^ The authors speculate that changes in patient selection for drug trials and/or improvements in supportive care could explain the differences.

The increasing duration of new treatments in recent years has been reported by others,^1^^,^^8^ which may be due to a transition from cytotoxic therapies administered in fixed durations, to monoclonal antibody strategies (including immunotherapy) and tyrosine kinase inhibitors which are administered continuously. For immunotherapy strategies, two examples in patients with unresectable or metastatic melanoma show that the duration of treatment in responders may be limited to 2 years (KEYNOTE-006 trial)^9^ or unlimited per protocol (CheckMate 067 trial).^10^ The setting and characteristics of patients receiving the treatment may also influence the duration of treatment, with data suggesting that real-world duration of treatment is much shorter than that of clinical trials, with treatment duration being 59% of that in clinical trials.^11^ However, to our knowledge, our work is the first one to examine the trend in duration of treatment for existing therapies within clinical trials. Given that patients enrolled in trials are generally highly selected, our results provide additional data in comparison with real-world outcomes. This information is valuable in determining relative efficacy. It is important to know, when a new drug is reported to be better than an older drug, that the efficacy was not artificially boosted by penalizing the old drug, thus introducing a bias in the study design, and casting doubts on the true results.

Why might the duration of treatment fall in subsequent studies? The bias may be present among sponsors, but the decision to continue a therapy is made by investigators. We hypothesize four potential mechanisms. Firstly, it is natural in medical oncology for the excitement of novel agents to dissipate over time. Sorafenib was once considered a transformational therapy for hepatocellular carcinoma, yet, as the years progressed, limitations of the product became more evident, and excitement for novel classes grew. By the time IMBrave150 tested the routine upfront administration of bevacizumab and atezolizumab in hepatocellular carcinoma, there was widespread enthusiasm for immunotherapy in this disease. Accordingly, some providers may not have pushed sorafenib on the control arm, as they once did in the SHARP study. Secondly, new second-line therapies emerge over time. At the time of the SHARP trial, there were no FDA-approved second-line therapies. Over time, regorafenib, ramucirumab, nivolumab, and cabozantinib emerged as second-line therapies, and some of these drugs (but not all) had demonstrated improvements in overall survival in subsequent lines. The availability of improved subsequent therapies may discourage investigators from pushing the initial medication. Thirdly, some trials may build in cross-over to the experimental drug as an enticement to investigators to deem a patient has progressed on the control arm. This may also be reflected in a discrepancy between investigator assessed and central review of PFS, with heavy PFS censoring in central review due to progression being called just short of the RECIST benchmark. Finally, the company may inadvertently nudge the investigator toward a climate where the goal of the study is considered to be the achievement of a statically significant result, and this may result in unconscious choices being made differently.^12^^,^^13^

The incentive to produce positive postmarketing trial results for drugs initially receiving accelerated approval could potentially bias researchers to discontinue treatment early in the control arm.^14^ We found evidence of both longer (alectinib in non-small-cell carcinoma) and shorter (cabozantinib in renal cell carcinoma) duration of treatment for drugs initially receiving accelerated approval being compared against a previously approved drug, so we cannot be sure whether postmarketing trial results influenced the duration of treatment for control-arm drugs.

There are several limitations to our study. Firstly, we had to impute values for duration of treatment, based on median PFS values, because these values were not always reported. The imputed values were supported by findings of a linear relationship between duration of treatment and PFS for >80% of studies that provided this information. Secondly, because multiple publications could be found for each drug, there was the potential to get slightly different PFS or duration-of-treatment values. In selecting studies and usable values, we used the values that corresponded with the data presented in the FDA label and used for FDA approval. Thirdly, we were not able to evaluate all instances because of lack of data (e.g. imatinib for chronic myeloid leukemia and dacarbazine for melanoma). Fourthly, there were relatively few instances (13% of approvals) that met our criteria (e.g. an approved drug was used as a comparator) since most approved drugs were tested against a placebo or no additional drug (55%). Fifthly, because of having few instances, we were not able to determine whether differences between the duration of treatment as the control and duration of treatment as the intervention were significantly different. As such, we can only determine whether the results were numerically different. Finally, it should be noted that setting up a trial may take >1 year, and the standard-of-care option may change during that time. Often these trials are continued, which may in part explain differences in control-arm choices.

### Conclusion

We found that when existing drugs are used as comparators, PFS and duration of treatment are shorter than when the same drugs were previously used when seeking FDA approval for a given indication. These results suggest that there is bias in how long a patient receives a given therapy, and this bias favors the newer therapy. Clinical trialists should seek to utilize methodology that reduces bias so that the relative efficacy of newer drugs can be objectively assessed.
